# Identification of Drug-Resistant Laterality in Bilateral Temporal Lobe Epilepsy

**DOI:** 10.7759/cureus.85081

**Published:** 2025-05-30

**Authors:** Hiroharu Suzuki, Takumi Mitsuhashi, Yasushi Iimura, Tetsuya Ueda, Kazuki Nishioka, Madoka Nakajima, Hidenori Sugano, Akihide Kondo

**Affiliations:** 1 Department of Neurosurgery, Juntendo University, Tokyo, JPN; 2 Department of Neurosurgery, Juntendo University Hospital, Tokyo, JPN; 3 Department of Neurosurgery, Sugano Neurosurgery Clinic, Tokyo, JPN

**Keywords:** antiseizure medication, epilepsy surgery, modulation index, phase-amplitude coupling, stereo-electroencephalography

## Abstract

Selecting the optimal resection side in bilateral temporal lobe epilepsy (BTLE) remains a challenge due to independent seizure origins in both temporal lobes. This study, therefore, aimed to evaluate whether antiseizure medication (ASM)-induced changes in the Modulation Index (MI), a measure of phase-amplitude coupling, could serve as a biomarker for determining the predominant laterality of drug resistance in BTLE.

A 35-year-old patient with drug-resistant BTLE underwent stereoelectroencephalography (SEEG) targeting bilateral temporal structures. To assess refractory epileptic focus, MI values were compared between the ASM-withdrawn and ASM-resumed states. The MI was calculated between high-frequency oscillations and 3-4 Hz slow-wave activity during interictal epochs.

Following ASM resumption, MI values significantly decreased in the right hippocampus and entorhinal cortex, while the left entorhinal cortex showed persistently elevated MI without significant reduction. These findings indicated a more refractory epileptic focus in the left temporal lobe. A left temporal lobectomy was performed, leading to seizure freedom for 37 months (International League Against Epilepsy (ILAE) Class 1), without changes in the ASM regimen.

ASM-induced MI changes in interictal SEEG may offer insights into lateralizing the more refractory temporal lobe in patients with BTLE and could potentially support resection side selection. However, further studies in larger cohorts are warranted to confirm its clinical utility and to establish standardized MI thresholds.

## Introduction

Surgical decision-making is particularly complex when considering resection in bilateral temporal lobe epilepsy (BTLE), as seizures can originate independently from each temporal lobe. Even with surgical intervention, the outcomes in BTLE cases remain suboptimal as compared to those of cases of unilateral temporal lobe epilepsy (TLE). A previous systematic review reported that patients with unilateral seizure onset achieved significantly higher postoperative seizure-freedom rates after surgical resection than those with bilateral involvement, highlighting the difficulty in accurately determining the dominant epileptogenic side in BTLE [1]. Residual epileptogenic activity in the contralateral hemisphere after surgery remains a major factor in seizure recurrence and further complicates surgical strategies.

Assessment of seizure laterality using intracranial electroencephalography (iEEG) is a key criterion for determining the resection site in cases of BTLE. A seizure laterality of 80% observed during iEEG monitoring has been suggested as a predictive marker of the dominant epileptogenic side and is a commonly used threshold [2-4]. However, the short duration of monitoring (one to two weeks) and sporadic nature of the seizures limit the reliability of this approach [5]. Additionally, chronic ambulatory electrocorticography studies have suggested that approximately eight months of monitoring are required to obtain a stable seizure laterality ratio in patients with drug-resistant BTLE, making this approach impractical for routine clinical decision-making [6].

Assessing the presence of a refractory epileptic focus as a marker of strong epileptogenicity may provide a more stable approach for determining the predominant laterality of epileptogenicity in BTLE [7]. Brain regions with persistent epileptic activity in the presence of antiseizure medication (ASM) administration may have strong epileptogenicity and may contribute more to seizure recurrence after surgery.

The Modulation Index (MI) is a parameter that reflects the degree of phase-amplitude coupling (PAC) between interictal high-frequency oscillations (HFOs; >80 Hz) and slow oscillations [8,9]. HFOs can be generated by both epileptogenic and functionally important cortical areas; however, their coupling with slow-wave activity may help distinguish pathological from physiological origins. Notably, epileptogenic HFOs tend to be preferentially coupled with slow oscillations in the 3-4 Hz range, whereas physiological HFOs are more often coupled with slower oscillations in the 0.5-1 Hz band during slow-wave sleep [10,11]. Therefore, MI calculated in the HFOs/3-4 Hz band serves as a marker of cortical excitability and reflects the severity of epileptiform activity, particularly spike-and-wave discharges.

Here, we report a case of a 35-year-old patient with BTLE who underwent stereoelectroencephalography (SEEG). We aimed to evaluate whether ASM-induced changes in the MI could act as a potential biomarker for determining the optimal resection side in cases of BTLE. Thus, we assessed the diagnostic utility of PAC between interictal HFOs and slow-wave activity, as quantified by the MI, and investigated whether MI changes in interictal SEEG recordings could identify the temporal lobe harboring the more refractory epileptic focus.

## Case presentation

Clinical course and surgical intervention

**Figure 1:**
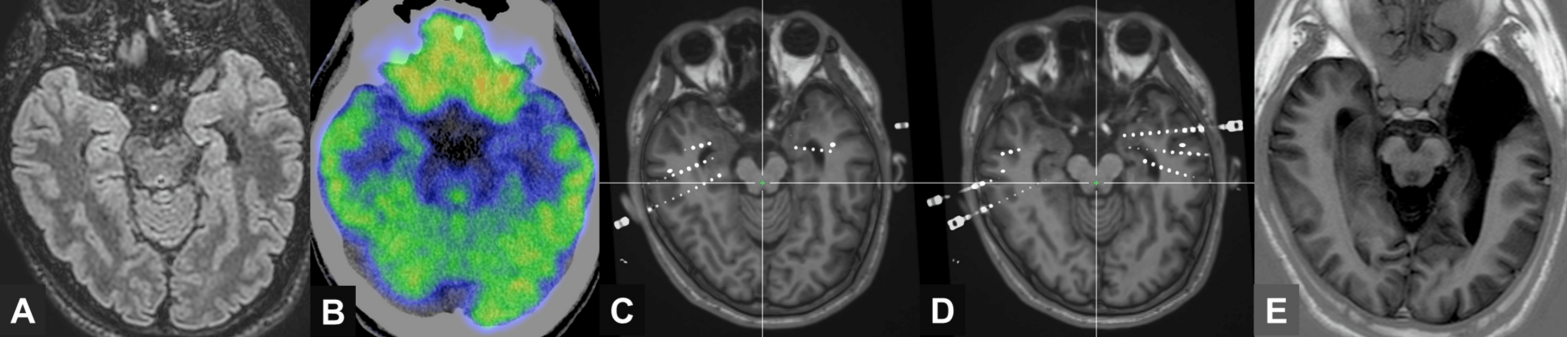
Multimodal imaging and electrode placement in a patient with bilateral temporal lobe epilepsy (A) Axial fluid-attenuated inversion recovery magnetic resonance imaging (MRI) showing bilateral hippocampal atrophy, which is more pronounced on the left side. (B) Interictal fluorodeoxyglucose-positron-emission tomography revealing hypometabolism in the left temporal lobe, including the mesial temporal structures. (C, D) Post-implantation axial MRI demonstrating the placement of stereoelectroencephalography electrodes targeting the hippocampus, entorhinal cortex, and lateral temporal cortex, bilaterally. (E) Postoperative MRI confirming the extent of left anterior temporal lobectomy, including hippocampal head resection.

A 35-year-old right-handed man with drug-resistant BTLE had experienced focal impaired awareness seizures (FIAS) since the age of 14. His treatment regimen included levetiracetam, carbamazepine, lamotrigine, sodium valproate, clonazepam, and vagus nerve stimulation (VNS). The VNS was programmed with an output current of 2.25 mA, frequency of 30 Hz, pulse width of 500 μs, and an on-off cycle of 30 seconds on and five minutes off. His habitual seizures typically began with an epigastric sensation, followed by impaired awareness and behavioral arrest. No clear lateralizing signs, such as unilateral dystonia, head version, or postictal hemiparesis, were observed. These FIAS occurred approximately once per week, and focal-to-bilateral tonic-clonic seizures occurred once every few months despite treatment with multiple ASMs and VNS. The Wechsler Memory Scale-Revised demonstrated a verbal memory score of 77, a visual memory score of 97, a general memory score of 80, an attention/concentration score of 90, and a delayed recall score of <50 before surgery. Magnetic resonance imaging revealed bilateral hippocampal atrophy, and fluorodeoxyglucose-positron-emission tomography (FDG-PET) revealed focal hypometabolism in the left anterior temporal lobe, including the hippocampus (Figures 1A-1B). Functional MRI (fMRI) for language mapping revealed bilateral language representation. A Wada test was not performed. Although memory lateralization was not directly assessed by fMRI, neuropsychological evaluation revealed verbal memory impairment, suggesting left hemisphere dominance for memory function.

During the three-day scalp video-electroencephalography (EEG) monitoring, all ASMs (levetiracetam, carbamazepine, lamotrigine, sodium valproate, and clonazepam) were discontinued, and VNS was turned off to enhance the likelihood of capturing habitual seizures and assessing lateralization. Subsequently, eight FIAS were recorded, with all ictal EEG traces showing right temporal rhythmic theta-delta discharges. Interictal epileptiform discharges (IEDs) were equally observed in both temporal lobes (T1, F7, T2, and F8 according to the International 10-20 system). Given these inconclusive findings, SEEG was performed to assess the seizure-onset zones and cortical excitability.

**Figure 2:**
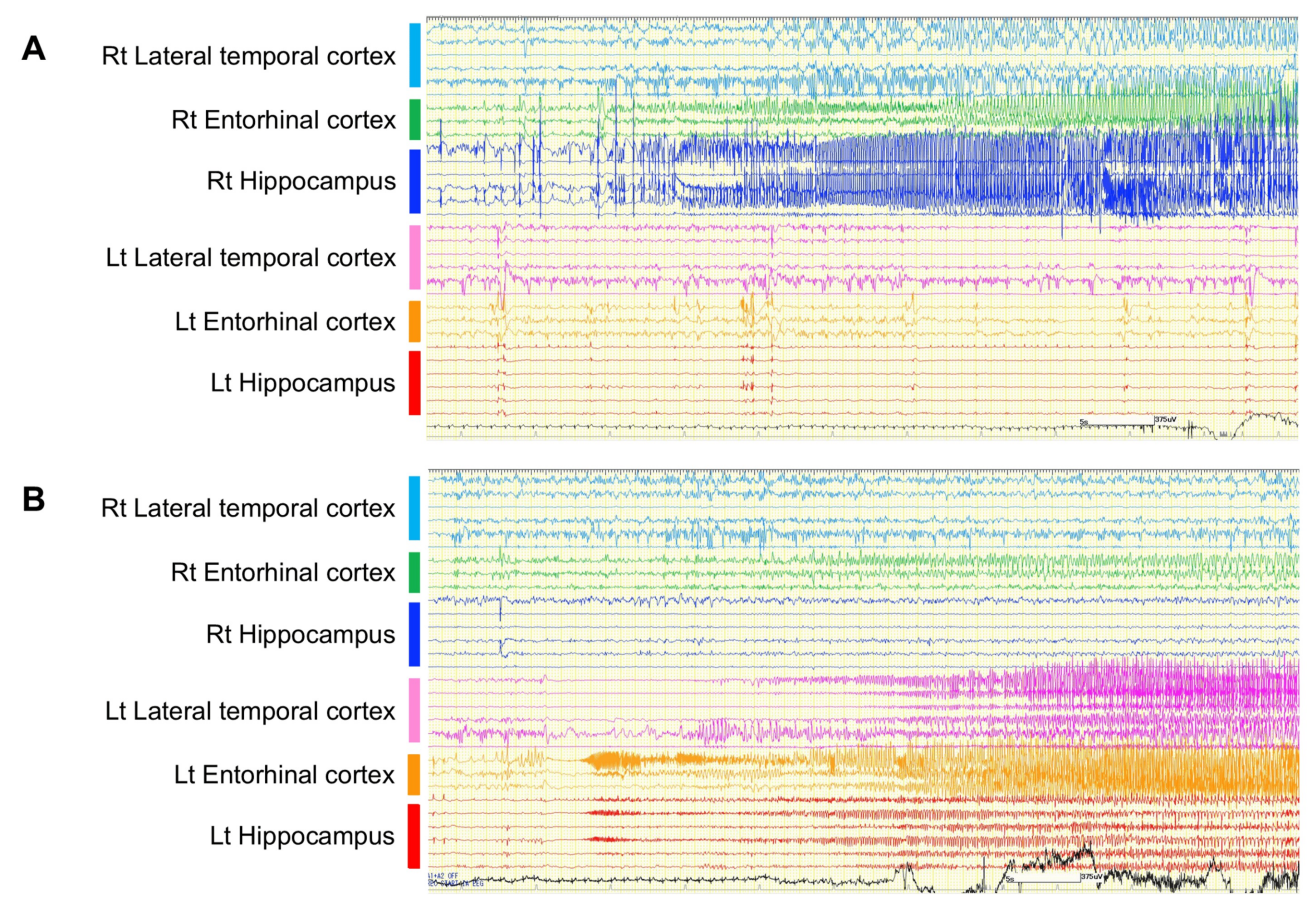
Representative SEEG recordings demonstrating bilateral seizure onsets (A) Seizure onset originating from the right hippocampus with propagation to the right lateral temporal neocortex. (B) Seizure onset originating in the left entorhinal cortex, with immediate spread to the left hippocampus, followed by propagation to the left lateral temporal neocortex. All traces are shown over a 60-second duration. Display settings: sensitivity 75 μV, time constant 0.3 s, high-frequency filter 120 Hz. SEEG: stereoelectroencephalography; Rt: right side; Lt: left side

Eight depth electrodes were implanted bilaterally using the ROSA ONE Brain System (Zimmer Biomet, Warsaw, IN, USA), targeting the mesial temporal structures (amygdala, hippocampal head, and hippocampal body) via the middle temporal gyrus and the entorhinal cortex via the superior temporal gyrus (Figures 1C-1D). During SEEG monitoring, the VNS was turned off to eliminate its potential influence on cortical activity. Prior to SEEG monitoring, serum levels of ASMs were obtained: levetiracetam 23.0 μg/mL, carbamazepine 10.4 μg/mL, lamotrigine 6.01 μg/mL, and sodium valproate 39.7 μg/mL. Clonazepam levels were not available, and post-discontinuation levels were not measured. During the first five days of SEEG recording, while all ASMs were discontinued, a total of 17 seizures were captured: four originating from the right hippocampus (Figure 2A), 10 from the right lateral temporal cortex, and three from the left entorhinal cortex. IEDs were equally present in both temporal lobes. After resuming lamotrigine alone (200 mg/day), three additional seizures originated from the left entorhinal cortex (Figure 2B), and the IEDs shifted to the left.

Based on these findings, left anterior temporal lobectomy, including atrophic hippocampal head resection, was performed (Figure 1E). The patient has subsequently remained seizure-free for 37 months (International League Against Epilepsy Class 1) on an unchanged preoperative ASM regimen. Histopathological examination of the resected hippocampus confirmed hippocampal sclerosis, while no overt structural abnormalities, including cortical dysplasia, were observed in the left entorhinal cortex.

SEEG data selection and PAC analysis

To assess the ASM-induced interictal cortical excitability changes and to identify the temporal lobe harboring the more refractory epileptic focus, PAC analysis was conducted on SEEG recordings.

Five 3-minute interictal phase epochs during slow-wave sleep were selected from the SEEG data recorded before and after ASM resumption. All five epochs were separated from each other and from seizures by a minimum of one hour. The MI was automatically calculated using the Phase-Amplitude Coupling Toolbox in MATLAB (MathWorks, Natick, MA, USA), following the method described by Iimura et al. [10] and Nonoda et al. [11].

Among the obtained MI values, we only analyzed those from the cortical contacts. Data were classified into three groups on each side: the lateral temporal cortex, entorhinal cortex, and hippocampus. The MI values before and after ASM resumption were calculated for each group, and the mean MI values were determined.

The Wilcoxon rank-sum test was used to determine the differences between the pre- and post-ASM resumption MI values, and the temporal lobe harboring the more refractory epileptic focus was identified. Statistical analyses were performed using IBM SPSS Statistics for Windows, Version 27 (Released 2021; IBM Corp., Armonk, New York, United States). Statistical significance was defined as p < 0.05.

MI for lateralization of drug resistance

**Figure 3:**
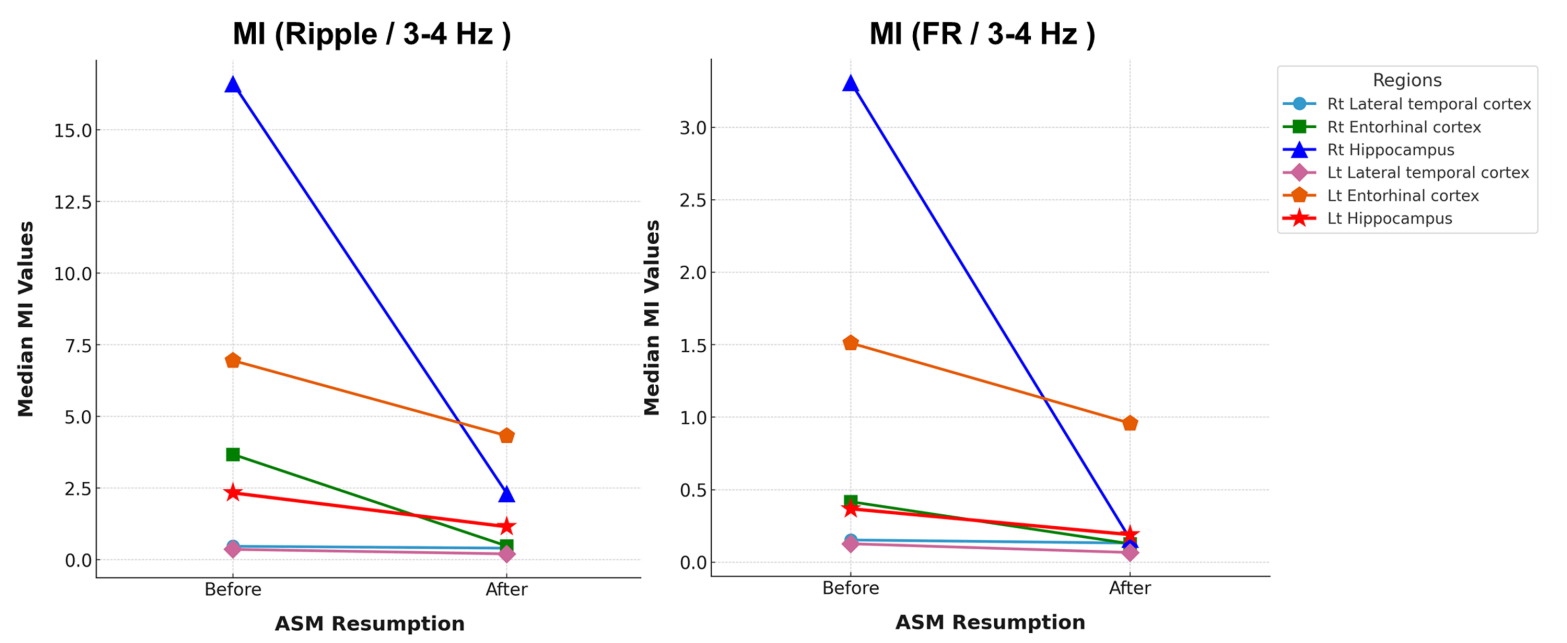
Modulation index changes in ripple and fast-ripple bands under the antiseizure medication resumption This figure illustrates the changes in the median Modulation Index (MI) values across six brain regions: the right-sided (Rt)/left-sided (Lt) lateral temporal cortex, Rt/Lt entorhinal cortex, and Rt/Lt hippocampus under before-antiseizure medication (ASM) and after-ASM resumption. (A) Ripple band (80-200 Hz/3-4 Hz), and (B) fast-ripple band (250-500 Hz/3-4 Hz). Each line represents a specific brain region. Right-sided regions are shown as follows: right lateral temporal cortex (light blue), right entorhinal cortex (green), right hippocampus (blue). Left-sided regions are shown as follows: left lateral temporal cortex (pink), left entorhinal cortex (orange), and left hippocampus (red). Overall, the MI values decreased following ASM resumption, indicating a general suppression of cortical excitability. Among all regions, the Lt entorhinal cortex retained the highest MI after ASM resumption, suggesting its relative resistance to ASM effects.

In MI Ripple/3-4 Hz analysis, ASM resumption led to a decrease in the MI values across all recorded regions, with statistically significant reductions in the right hippocampus (p = 0.02, mean MI Ripple/3-4 Hz values: from 16.62 to 2.306), right entorhinal cortex (p = 0.02, from 3.674 to 0.477), left hippocampus (p = 0.004, from 2.331 to 1.150), and left lateral temporal cortex (p = 0.01, from 0.360 to 0.203). In contrast, the reductions in the right lateral temporal cortex (p = 0.99) and left entorhinal cortex (p = 0.29) were not significant (Figure 3 and Table 1).

**Table 1:** Modulation Index values before and after antiseizure medication resumption across six temporal regions in two phase-amplitude coupling frequency bands Mean modulation index (MI) values are shown for Ripple (80-200 Hz)/3-4 Hz and Fast Ripple (FR, 250-500 Hz)/3-4 Hz phase-amplitude coupling, measured from interictal SEEG recordings. MI values were compared before and after antiseizure medication (ASM) resumption. Statistically significant p-values (*: < 0.05) are highlighted for each region, indicating ASM-induced suppression of cortical excitability. Notably, the left entorhinal cortex maintained high MI values without significant reduction in either frequency band, suggesting the presence of a refractory epileptic focus.

	Ripple/3-4Hz			FR/3-4Hz		
	MI Before ASM resumption	MI After ASM resumption	p-value	MI Before ASM resumption	MI After ASM resumption	p-value
Rt Lateral temporal cortex	0.468	0.403	0.99	0.153	0.132	0.74
Rt Entorhinal cortex	3.674	0.477	0.02*	0.417	0.127	0.02*
Rt Hippocampus	16.62	2.306	0.02*	3.310	0.158	0.01*
Lt Lateral temporal cortex	0.360	0.203	0.01*	0.127	0.067	0.08
Lt Entorhinal cortex	6.951	4.325	0.29	1.511	0.958	0.29
Lt Hippocampus	2.331	1.150	0.004*	0.368	0.190	0.02*

Despite the overall reduction in the MI values, the left entorhinal cortex exhibited the highest MI values after ASM resumption (MI Ripple/3-4 Hz 4.325), with no statistically significant reduction (p = 0.29), suggesting the presence of a refractory epileptic focus in this region.

Similarly, in the MI Fast Ripple (FR)/3-4 Hz analysis, MI values decreased across all regions, with significant reductions in the right hippocampus (p = 0.01, MI FR/3-4 Hz values from 3.310 to 0.158), right entorhinal cortex (p = 0.02, from 0.417 to 0.127), and left hippocampus (p = 0.02, from 0.368 to 0.190). The decrease in the left lateral temporal cortex (p = 0.08, from 0.127 to 0.067) showed a trend toward significance, but did not reach statistical significance, while changes in the right lateral temporal cortex (p = 0.74) and left entorhinal cortex (p = 0.29) were not statistically significant.

After ASM resumption, the left entorhinal cortex (MI FR/3-4 Hz 0.958) maintained a relatively high MI value without showing a significant reduction (p = 0.29), indicating the presence of a persistent refractory epileptic focus.

## Discussion

In this case of BTLE, our results demonstrated global suppression of the MI after ASM resumption, with significant reductions in the hippocampus and entorhinal cortex, particularly on the right side. The left entorhinal cortex exhibited persistent MI elevation after ASM was resumed, which suggested strong epileptogenicity on this side and supported its selection for resection. These findings suggest that ASM-induced changes in MI may serve as biomarkers for determining the optimal resection side by identifying the temporal lobe containing the more refractory epileptic focus in cases of BTLE.

Seizure count-based laterality assessments are limited by the need to capture multiple seizures within a restricted monitoring period [12,13]. Seizures are inherently sporadic, and short monitoring durations in clinical settings may not always be sufficient to establish a stable laterality pattern, particularly in cases of BTLE, where both temporal lobes exhibit seizure activity independently [6,14]. In our case, although scalp EEG demonstrated ictal discharges predominantly in the right temporal lobe and interictal discharges in both hemispheres, these findings were inconclusive for lateralization. While initial seizures on SEEG were more frequent in the right temporal lobe, the seizure onset shifted to the left entorhinal cortex after the resumption of ASM, illustrating the dynamic nature of seizure lateralization in BTLE. These findings highlight that even when both scalp EEG and intracranial EEG are evaluated together, dynamic changes in seizure laterality can obscure clear lateralization, potentially leading to misjudgment of the resection side. This underscores the clinical need for additional interictal biomarkers to support surgical decision-making.

Our approach addresses this challenge by applying interictal PAC analysis to SEEG data using the MI as a quantitative measure, rather than relying solely on seizure counts. This method allows a more consistent and reproducible evaluation of highly epileptogenic regions within a shorter monitoring period. Interictal PAC-based analysis on SEEG data does not replace seizure count-based laterality assessment but rather complements it while reducing the risk of misinterpretation in cases with fluctuating seizure laterality. While PAC analysis has been explored in scalp EEG in previous studies [15], we chose not to apply it in this case. The spatial resolution and signal-to-noise ratio of scalp EEG are generally insufficient for detecting HFOs and their PAC [16]. Furthermore, the present study focused on evaluating SEEG-based PAC analysis in the context of non-lateralizing scalp EEG findings and thus did not extend PAC analysis to scalp EEG recordings.

EEG-based analysis of ASM effects, known as pharmaco-EEG, has been widely utilized in clinical neuropharmacology to study ASM-induced modulation of brain activity [17]. In epilepsy research, pharmaco-EEG allows assessment of the effects of ASM on cortical excitability and network functions. Previous studies have demonstrated that specific ASMs alter cortical activity and connectivity patterns, reflecting their impact on seizure control. Patients with TLE demonstrated improved seizure control as levetiracetam reduces excessive connectivity within epileptogenic networks while enhancing long-range cortical synchronization [18]. Similarly, a previous study reported that brivaracetam increased theta phase-locking value (PLV) connectivity in responders, bringing their connectivity metrics closer to those of healthy controls, whereas non-responders exhibited persistent hyper-connectivity, indicating ongoing epileptogenic activity, despite ASM administration [19].

Building upon these findings, our study demonstrated that ASM-induced MI changes can serve as biomarkers for lateralizing the more refractory epileptic focus in BTLE. The observed global reduction in MI after ASM resumption suggests that the regions responding to lamotrigine exhibit suppressed pathological high-frequency activity. In contrast, the left entorhinal cortex retained a relatively high MI after ASM resumption, showing no significant MI reduction, which supported its role as a highly refractory epileptic focus. These findings reinforce the potential value of integrating PAC analysis into clinical decision-making. Notably, despite the persistently elevated MI in the left entorhinal cortex after ASM resumption, histopathological examination revealed no structural abnormalities. This dissociation suggests that the elevated MI may indicate functionally active epileptogenic regions without corresponding histopathological changes. Identifying cortical hyperexcitability that persists despite ASM administration may offer an additional criterion for optimizing surgical strategies for drug-resistant epilepsy.

This study has several limitations that should be considered. First, as this was a single-case study, the findings cannot be generalized. In this case, the decision to perform a left temporal lobectomy was based on a comprehensive evaluation of the MRI, PET, neuropsychological assessments, and SEEG findings. While the ASM-induced MI changes were not the sole determinant, it provided complementary insights into cortical excitability and refractory epileptogenicity. Second, standardized MI thresholds for clinical application remain undefined, which limits the immediate applicability of this method across different institutions or patient populations. Moreover, we did not evaluate the potential influence of physiological or environmental factors such as time-dependent fluctuations, sleep-wake states, stress, or external conditions, which may also affect MI values. Future research should aim to validate MI as a biomarker in larger cohorts, compare it with other quantitative EEG metrics such as PLV, and evaluate its utility in non-resective therapies such as responsive neurostimulation.

## Conclusions

This study suggested that ASM-induced changes in the MI may serve as biomarkers for determining the predominant laterality of the refractory epileptic focus in patients with BTLE. By evaluating cortical excitability changes under the influence of ASM, rather than relying solely on seizure count-based laterality assessments, surgical decision-making can be refined and surgical outcomes optimized in cases of BTLE. Future studies should aim to validate this approach in larger cohorts and define clinically applicable MI thresholds.
